# Supplementary material for: Brain-wide correspondence of neuronal epigenomics and distant projections
Source: Nature. 2023 Dec 13;624(7991):355–65. doi: 10.1038/s41586-023-06823-w (PMC10719087; doi:10.1038/s41586-023-06823-w)
Supplement: Supplementary file 1 — Reporting Summary [file 41586_2023_6823_MOESM1_ESM.pdf]

nature portfolio

Corresponding author(s): Edward M. Callaway, Joseph R. Ecker

Last updated by author(s): Oct 30, 2023

Reporting Summary

Nature Portfolio wishes to improve the reproducibility of the work that we publish. This form provides structure for consistency and transparency in reporting. For further information on Nature Portfolio policies, see our Editorial Policies and the Editorial Policy Checklist.

Statistics

For all statistical analyses, confirm that the following items are present in the figure legend, table legend, main text, or Methods section.

n/a	Confirmed	
☐	☒	The exact sample size (n) for each experimental group/condition, given as a discrete number and unit of measurement
☐	☒	A statement on whether measurements were taken from distinct samples or whether the same sample was measured repeatedly
☐	☒	The statistical test(s) used AND whether they are one- or two-sided<br>*Only common tests should be described solely by name; describe more complex techniques in the Methods section.*
☒	☐	A description of all covariates tested
☐	☒	A description of any assumptions or corrections, such as tests of normality and adjustment for multiple comparisons
☐	☒	A full description of the statistical parameters including central tendency (e.g. means) or other basic estimates (e.g. regression coefficient) AND variation (e.g. standard deviation) or associated estimates of uncertainty (e.g. confidence intervals)
☐	☒	For null hypothesis testing, the test statistic (e.g. F, t, r) with confidence intervals, effect sizes, degrees of freedom and P value noted<br>*Give P values as exact values whenever suitable.*
☒	☐	For Bayesian analysis, information on the choice of priors and Markov chain Monte Carlo settings
☒	☐	For hierarchical and complex designs, identification of the appropriate level for tests and full reporting of outcomes
☐	☒	Estimates of effect sizes (e.g. Cohen's d, Pearson's r), indicating how they were calculated

Our web collection on statistics for biologists contains articles on many of the points above.

Software and code

Policy information about availability of computer code

Data collection	BD Influx Sortware v1.2.0.142 (flow cytometry), Freedom EVOware v2.7 (library preparation), Illumina MiSeq control software v3.1.0.13 and NovaSeq 6000 control software v1.6.0/RTA v3.4.4 (sequencing), Olympus cellSens Dimension 1.8 (image acquisition)
Data analysis	System: python=3.7.12, skypilot==1.0.0.dev0;<br>Mapping: yap=1.5.11, cutadapt=2.10, bismark=0.20.0, bowtie2=2.3.5, picard=2.18, samtools=1.9;<br>Analysis: allcools=1.0.17, anndata=0.8.0, scanpy=1.9.1, zarr=2.12.0, bedtools=2.30.0, scikit-learn=1.0.2, h5py=3.7.0, hdf5=1.12.2, htslib=1.16, matplotlib-base=3.5.2, harmonypy=0.0.9, qnorm=0.8.1, wmb=0.1.36;<br>Other code are available on https://github.com/zhoujt1994/EpiRetroSeq2023.git

For manuscripts utilizing custom algorithms or software that are central to the research but not yet described in published literature, software must be made available to editors and reviewers. We strongly encourage code deposition in a community repository (e.g. GitHub). See the Nature Portfolio guidelines for submitting code & software for further information.

Data

Policy information about availability of data

All manuscripts must include a data availability statement. This statement should provide the following information, where applicable:

- Accession codes, unique identifiers, or web links for publicly available datasets
- A description of any restrictions on data availability
- For clinical datasets or third party data, please ensure that the statement adheres to our policy

Raw and processed data are also available at GEO under accession code GSE230782. Processed data can be explored on our web portal: http://neomorph.salk.edu/epiretro. Other datasets used in this study include scRNA-seq (https://portal.brain-map.org/atlases-and-data/bkp/abc-atlas), snmC-seq and MERFISH (https://mousebrain.salk.edu/download), Act-Seq (DOI 10.17632/ypx3sw2f7c.3), and Retro-Seq (GSE133912). The mm10 genome was downloaded from https://hgdownload.soe.ucsc.edu/goldenPath/mm10/bigZips/.

Research involving human participants, their data, or biological material

Policy information about studies with human participants or human data. See also policy information about sex, gender (identity/presentation), and sexual orientation and race, ethnicity and racism.

Reporting on sex and gender	N/A
Reporting on race, ethnicity, or other socially relevant groupings	N/A
Population characteristics	N/A
Recruitment	N/A
Ethics oversight	N/A

Note that full information on the approval of the study protocol must also be provided in the manuscript.

Field-specific reporting

Please select the one below that is the best fit for your research. If you are not sure, read the appropriate sections before making your selection.

☒ Life sciences ☐ Behavioural & social sciences ☐ Ecological, evolutionary & environmental sciences

For a reference copy of the document with all sections, see nature.com/documents/nr-reporting-summary-flat.pdf

Life sciences study design

All studies must disclose on these points even when the disclosure is negative.

Sample size	No sample size calculation was performed. Number of cells from each experiment after QC were reported in Supplementary Table 3. The sample size allowed us to obtain high coverage methylomes for each projection, and confidently identify projection neuron enriched clusters.
Data exclusions	We imaged the live tissue and closely inspected every injection site to ensure that the injection location was as intended and off-target injections were eliminated.<br>Poor quality nuclei were excluded from downstream analyses through four steps of quality controls (QCs) described in Methods.
Replication	At least 2 male and 2 female mice were injected with AAV-retro-Cre for each projection target. Male and female samples were pooled separately for nuclei preparation. Nuclei collected from the male and female pool were used as biological replicates in the downstream analyses. The consistency of projection enriched clusters between biological replicates are reported in Extended Data Fig. 5. The comparison of results from biological replicates and computational replicates are shown in Extended Data Fig. 3 and discussed in "Quantification of projection neuron difference with AUROC" in Methods.
Randomization	Animals used for injections into each brain area were selected at random. FACS sorted cells selected for sequencing were randomized during the sorting process.
Blinding	Technicians doing nuclei preps and snmC-seq analyses were blind to the injection sites used for each sample.

Reporting for specific materials, systems and methods

We require information from authors about some types of materials, experimental systems and methods used in many studies. Here, indicate whether each material, system or method listed is relevant to your study. If you are not sure if a list item applies to your research, read the appropriate section before selecting a response.

Materials & experimental systems

n/a	Involved in the study
☐	☒ Antibodies
☒	☐ Eukaryotic cell lines
☒	☐ Palaeontology and archaeology
☐	☒ Animals and other organisms
☒	☐ Clinical data
☒	☐ Dual use research of concern
☒	☐ Plants

Methods

n/a	Involved in the study
☒	☐ ChIP-seq
☐	☒ Flow cytometry
☒	☐ MRI-based neuroimaging

Antibodies

Antibodies used	anti-GFP antibody, dilution: 1:500, Alexa Fluor 488 (Invitrogen, A-21311)<br>anti-NeuN antibody, clone A60, dilution: 1:300, EMD Millipore MAB377 conjugated with Alexa Fluor 647 (Invitrogen A20173)
Validation	Anti-NeuN antibodies have been previously published for use in immunohistochemistry and flow cytometry experiments (PMID: 23828890, 26087164). Anti-GFP antibody has been validated in Kim et al. Neuron 2020 (PMID: 32396852).

Animals and other research organisms

Policy information about studies involving animals; ARRIVE guidelines recommended for reporting animal research, and Sex and Gender in Research

Laboratory animals	The knock-in mouse line, R26R-CAG-loxp-stop-loxp-Sun1-sfGFP-Myc (INTACT) used in Epi-Retro-Seq was maintained on a C57BL/6J background. Adult male and female INTACT mice were used for the retrograde labeling experiments. Animals were housed in an AAALAC accredited facility at the Salk Institute. Lighting was controlled on a 12 hour light/12 hour dark cycle. Temperature was monitored and adjusted in accordance with Guide for the Care and Use of Laboratory Animals. Humidity was not controlled but monitored. Because all air coming in is 100% fresh air (not re-circulated), humidity in the animal facilities is approximately the same as the outside ambient air. San Diego averages 40-60% humidity year-round. Animals were 35-54 days old at the time of surgery for viral vector injections, were sacrificed 13-17 days later, and were 50-70 days old on the day of dissection.<br>56-63 day old, C57BL/6J "wild-type" mice were used for MERFISH experiments.
Wild animals	N/A
Reporting on sex	The sample sizes for different sexes are reported in Supplementary Table 3. The consistency of projection enriched clusters between sexes are reported in Extended Data Fig. 5. Different sexes are used to split training and testing sets and the performances were compared with random split in Extended Data Fig. 3.
Field-collected samples	The study did not involve samples collected from the field.
Ethics oversight	All experimental procedures using live animals were approved by the Salk Institute Animal Care and Use Committee.

Note that full information on the approval of the study protocol must also be provided in the manuscript.

Plants

Seed stocks	N/A
Novel plant genotypes	N/A
Authentication	N/A

Flow Cytometry

Plots

Confirm that:

☒ The axis labels state the marker and fluorochrome used (e.g. CD4-FITC).

☒ The axis scales are clearly visible. Include numbers along axes only for bottom left plot of group (a 'group' is an analysis of identical markers).

☒ All plots are contour plots with outliers or pseudocolor plots.

☒ A numerical value for number of cells or percentage (with statistics) is provided.

Methodology

Sample preparation	Manually dissected mouse brain samples were snap-frozen on dry ice and stored at -80 °C. Prior to nuclei preparation, for each projection, samples from 2 males and 2 females were pooled separately as biological replicates. The frozen brain tissues were transferred to a pre-chiled 2-mL dounce homogenizer with 1 mL ice-cold NIM buffer (0.25M sucrose, 25mM KCl, 5mM MgCl2, 10mM Tris-HCl (pH7.4), 1mM DTT (Sigma 646563), 10µl of protease inhibitor (Sigma P8340)), with 0.1% Triton X-100 and 5µM Hoechst 33342 (Invitrogen H3570), and gently homogenized on ice with the pre-chilled pestle 10-15 times. The homogenate was transferred to pre-chilled microcentrifuge tubes and centrifuged at 1000 rcf for 8 min at 4 °C to pellet the nuclei. The pellet was resuspended in 1 mL ice-cold NIM buffer, and again centrifuged at 1000 rcf for 8 min at 4 °C. The pellet was then resuspended in 450 µL of ice-cold NSB buffer (0.25M sucrose, 5mM MgCl2, 10mM Tris-HCl (pH7.4), 1mM DTT, 9ul of Protease inhibitor), and filtered through 40µM cell strainer. The filtered nuclei suspension was incubated on ice for at least 30 minutes with 50µl of nuclease-free BSA for at least 10 minutes, then incubated with GFP antibody, Alexa Fluor 488 (Invitrogen, A-21311) and anti-NeuN antibody (EMD Millipore MAB377) conjugated with Alexa Fluor 647 (Invitrogen A20173). GFP+/NeuN+ single nuclei were isolated using fluorescence-activated nuclei sorting (FANS) on a BD Influx sorter with 100µm nozzle, and sorted into 384-well plates preloaded with 2µl of digestion buffer for snmC-seq215 (20 mL digestion buffer consists of 10 mL M-digestion buffer (2×, Zymo D5021-9), 1 ml Proteinase K (20 mg, Zymo D3001-2-20), 9 mL water, and 10 µL unmethylated lambda DNA (100 pg/µL, Promega, D1521)). The collected plates were incubated at 50 °C for 20 minutes then stored at -20 °C.
Instrument	BD Influx
Software	BD Influx Sortware v1.2.0.142
Cell population abundance	We sorted NeuN-positive and GFP-positive nuclei.
Gating strategy	Intact nuclei were first discriminated from debris by virtue of their bright DNA labeling (Hoechst Height signal) followed by light scattering profiles (Forward Scatter (FSC) Height vs Side Scatter (SSC) Height). Events with high Pulse Width measurements for FSC and SSC were then excluded as aggregates. Next, NeuN-AlexaFluor 647 labelled neuronal nuclei were selected ("*670/30 640" Height) from which GFP positive nuclei were sorted ("*530/40 488" Height).

☒ Tick this box to confirm that a figure exemplifying the gating strategy is provided in the Supplementary Information.
